# Crystal structure of bis­[tris­(1,10-phenanthroline-κ^2^ *N*,*N*′)cobalt(II)] tetra­nitrate *N*,*N*′-(1,4-phenyl­enedicarbon­yl)diglycine solvate octa­hydrate

**DOI:** 10.1107/S2056989015013006

**Published:** 2015-07-11

**Authors:** Niels-Patrick Pook, Philipp Hentrich, Mimoza Gjikaj

**Affiliations:** a Institute of Inorganic and Analytical Chemistry, Clausthal University of Technology, Paul-Ernst-Strasse 4, D-38678 Clausthal-Zellerfeld, Germany

**Keywords:** crystal structure, cobalt(II) complex, *N*,*N*′-(1,4-phenyl­enedicarbon­yl)diglycine, phenanthroline ligand, supra­molecular inter­actions

## Abstract

The complex cation of the title compound, [Co(C_12_H_8_N_2_)_3_]_2_(NO_3_)_4_·C_12_H_12_N_2_O_6_·8H_2_O, contains a Co^II^ atom with a distorted octa­hedral coordination environment defined by six N atoms from three bidentate 1,10-phenanthroline ligands. The asymmetric unit of the title compound is completed by one-half of the *N*,*N*′-(1,4-phenyl­enedicarbon­yl)diglycine solvent mol­ecule, which is located on a centre of inversion, by two nitrate counter-anions and four solvent water mol­ecules. Two [Co(C_12_H_8_N_2_)_3_]^2+^ cations are connected through C—H⋯O contacts and through lone-pair⋯π inter­actions involving the non-coordinating *N*,*N*′-(1,4-phenyl­enedicarbon­yl)diglycine and phenanthroline mol­ecules. The different aromatic ring systems are involved in π–π stacking and C—H⋯π inter­actions, with centroid-to-centroid distances in the range 3.7094 (8)–3.9973 (9) Å. The crystal structure is stabilized by further anion⋯π inter­actions and C—H⋯O contacts, as well as O—H⋯O and N—H⋯O hydrogen bonds between water mol­ecules, the non-coordinating nitrate anions, *N*,*N*′-(1,4-phenyl­enedicarbon­yl)diglycine and phenanthroline mol­ecules. These non-covalent inter­actions give rise to a three-dimensional supra­molecular network.

## Abstract 2

The complex cation of the title compound includes one Co^II^ atom with a distorted octa­hedral coordination environment defined by six N atoms from three bidentate phenanthroline ligands. The non-coordinating *N*,*N*′-(1,4-phenyl­enedicarbon­yl)diglycine ligand links the cationic building blocks *via* C—H⋯O contacts and through lone-pair⋯π inter­actions. Further observed non-covalent inter­actions contribute to the consolidation of the supra­molecular network.

## Chemical context   

In the past decades, the focus on metal-organic complexes which form coordination polymers of different dimensions has drawn much attention due to their inter­esting structures and physical and chemical properties. Application fields for these materials are in catalysis, in gas storage (Kitagawa *et al.*, 2004 ▸), luminescence (Allendorf *et al.*, 2015 ▸) and very recently as scintillation materials (Allendorf *et al.*, 2009 ▸; Doty *et al.*, 2009 ▸; Perry *et al.*, 2012 ▸). The structures of coordination polymers (Leong & Vittal, 2011 ▸; Yamada *et al.*, 2013 ▸) often show various non-covalent inter­molecular inter­actions and forces, and therefore are intimately connected with the field of supra­molecular chemistry (Schneider, 2009 ▸) and self-assembly (Cook *et al.*, 2013 ▸). Such non-covalent inter­actions are also of utmost importance in biological macromolecules like DNA, RNA and proteins (Salonen *et al.*, 2011 ▸). They are typically observed in biochemical reactions as protein–ligand recognitions and are partly utilized in drug design (Meyer *et al.*, 2003 ▸). Apart from classical and non-classical hydrogen bonding of the types O–H⋯O, N—H⋯O and C—H⋯O, respectively, different π-inter­actions of aromatic rings such as π–π stacking, C—H⋯π, ion⋯π and lone-pair⋯π play a crucial role in the assembly of metal-organic polymers. Nitro­gen-containing heterocycles like bi­pyridine and phenanthroline are metal-coordinating, electron-deficient aromatic systems and predestined for π–π stacking as π-acceptors (Janiak, 2000 ▸). In addition, π-donor⋯acceptor functions in different parts of an aromatic mol­ecule can lead to remarkable properties (Albrecht *et al.*, 2010 ▸).
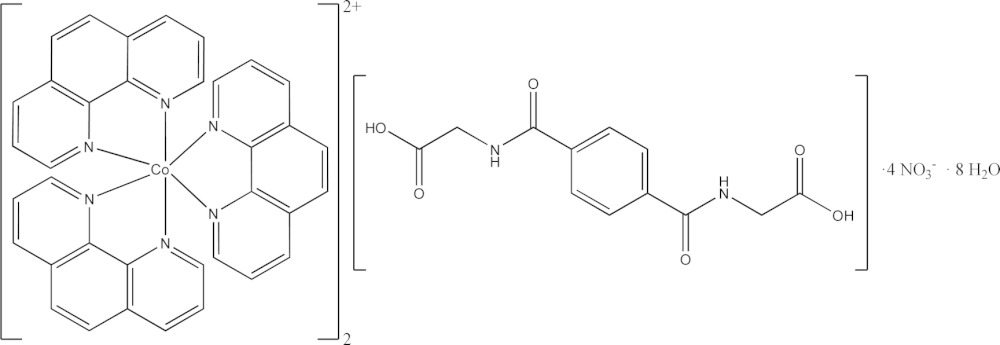


In previously synthesized transition metal complexes with *N*,*N*′-(1,4-phenyl­enedicarbon­yl)diglycine as metal-linking ligand, zigzag chains are formed, constructing inter­penetrating networks (see *Database survey*). In our synthetic approach, we offer such systems another electron-deficient bidentate aromatic ring system like phenanthroline or bi­pyridine in order to block parts of the coordination sphere of the metal atoms so that these zigzag chains are truncated or not formed at all. Thus, an alternative route for the resultant system lies in the use of the offered π-inter­action possibilities as well as in stacking inter­actions as a new linking mode. Recently, we have described the inter­actions of a cobalt(III) bi­pyridine complex with supra­molecular synthons (Pook *et al.*, 2014 ▸) as well as a precursor material (Pook *et al.*, 2013 ▸) that both contain *N*,*N*′-(1,4-phenyl­enedicarbon­yl)diglycine. The chosen ligand *N*,*N*′-(1,4-phenyl­enedicarbon­yl)diglycine is a relatively rigid mol­ecule with one *sp* ^3^-hybridized methyl­ene carbon atom that allows the acid moiety to rotate. Moreover, this ligand simultaneously possesses several coordination sites through the carb­oxy­lic group and the oxygen atom of the amide group. These functional groups can also be involved in hydrogen bonding and *D*—H⋯π inter­actions.

In the present contribution we have determined the structure of a novel cobalt(II) coordination polymer with a non-coordinating *N*,*N*′-(1,4-phenyl­enedicarbon­yl)diglycine solvent molecule linking two tris­(phenanthroline)cobalt(II) cationic building blocks *via* the mentioned non-classical inter­actions.

## Structural commentary   

**Figure 1:**
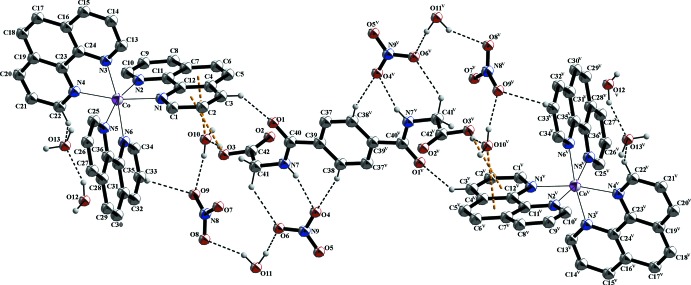
The mol­ecular entities of the title structure with atom labels and displacement ellipsoids of non-H atoms drawn at the 50% probability level. Dashed lines indicate N—H⋯O and O—H⋯O hydrogen bonds, as well as lone-pair⋯π inter­actions (see Table 1 ▸ for details). [Symmetry code: (v) −*x*, −*y* + 2, −*z*.]

The mol­ecular entities (Fig. 1 ▸) of the title compound include one Co^II^ complex cation in which three bidentate phenanthroline ligands define a distorted octa­hedral coordination sphere. Distances and angles of this rather common cationic species, [Co(C_12_H_8_N_2_)_3_]^2+^, are well within expected ranges and are comparable to those found in the literature (Li *et al.*, 2011 ▸; Geraghty *et al.*, 1999 ▸). A crystallographic center of inversion is located at the centroid of the protonated and non-coordin­ating *N*,*N*′-(1,4-phenyl­enedicarbon­yl)diglycine molecule. The asymmetric unit is completed by two non-coordinating nitrate counter-anions and four solvent water mol­ecules. The *N*,*N*′-(1,4-phenyl­enedicarbon­yl)diglycine mol­ecule links two complex tris­(phenanthroline-κ^2^N,N′)cobalt(II) cations *via* lone-pair⋯π inter­actions involving the carb­oxy­lic acid function and the phenanthroline aromatic system as well as C—H⋯O contacts between the oxygen atom of the amide group and one phenanthroline ligand. Moreover, π–π stacking inter­actions between different aromatic ring systems and C—H⋯π as well as O—H⋯O and N—H⋯O hydrogen bonding are observed and consolidate an extensive three-dimensional supra­molecular network.

## Supra­molecular features   

**Figure 2:**
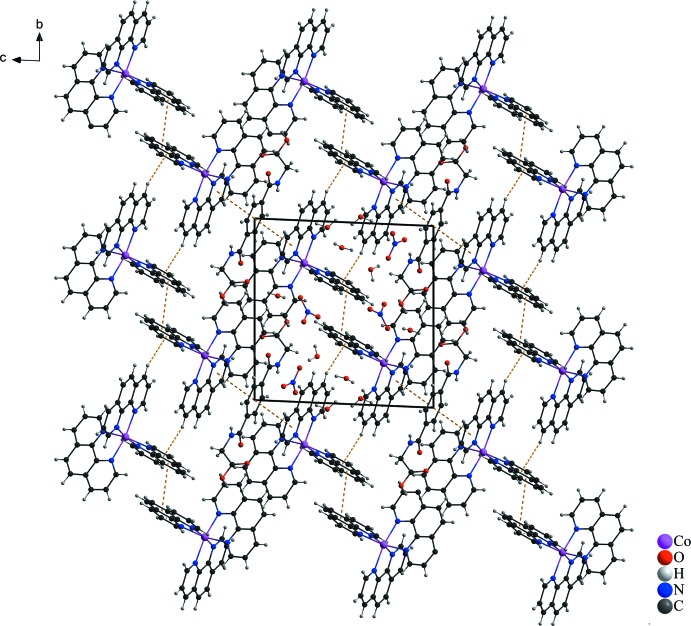
The crystal packing of the title structure in a view along the *a* axis. Selected π–π stacking and C—H⋯π inter­actions are shown as dashed lines.

**Figure 3:**
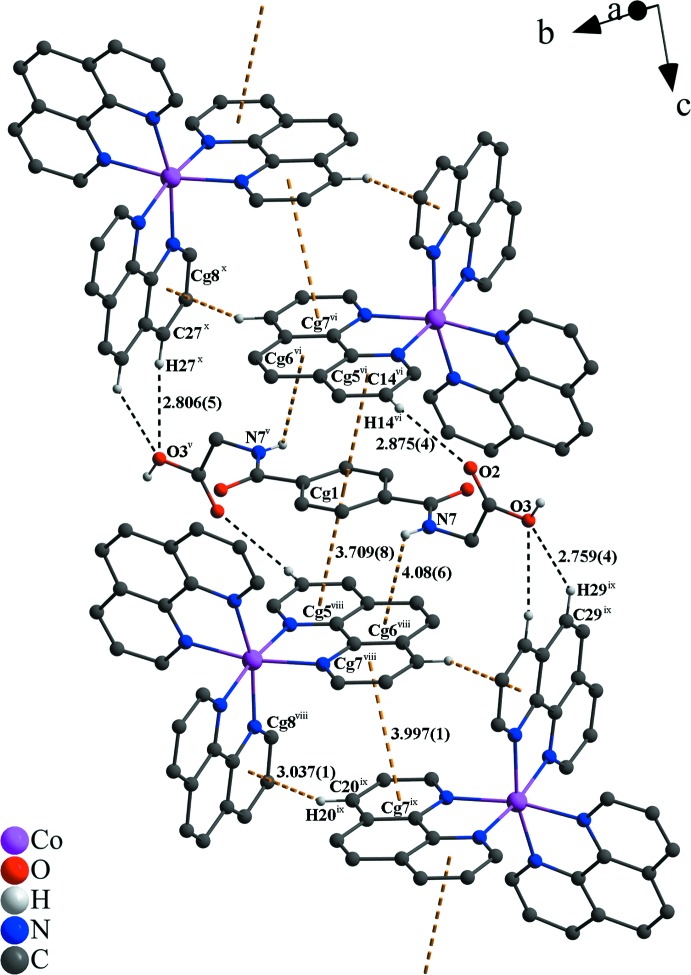
In the crystal packing, different non-covalent inter­actions such as C—H⋯O contacts and π–π stacking, N—H⋯π and C—H⋯π inter­actions between the aromatic moieties are present (dashed lines; distances are given in Å). [Symmetry codes: (v) −*x*, −*y* + 2, −*z*; (vi) −*x*, −*y* + 1, −*z*; (viii) *x*, *y* + 1, *z*; (ix) −*x*, −*y* + 1, −*z* + 1; (x) *x*, *y* + 1, *z* − 1.]

**Figure 4:**
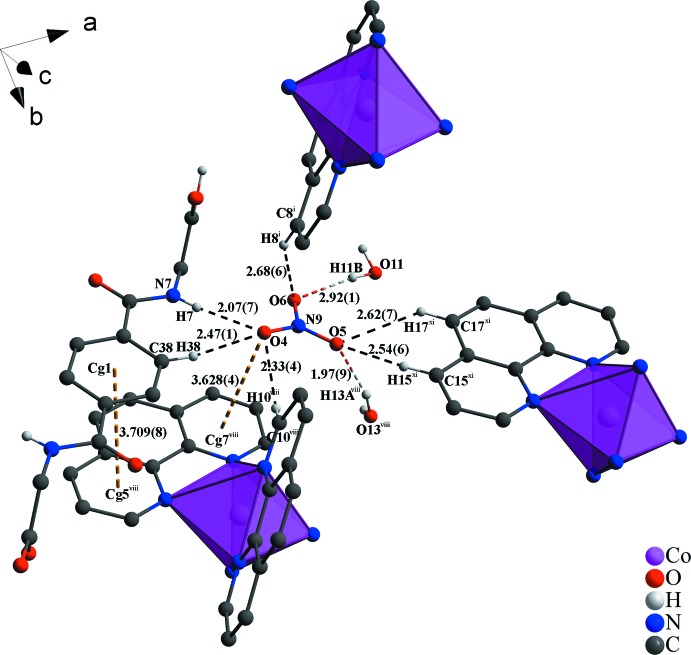
View of the anion⋯π inter­action and the extended network of O—H⋯O and C—H⋯O hydrogen bonds with the embedded non-coordinating nitrate anion (N9/O4–O6) as well as π–π stacking. O—H⋯O contacts are indicated by red–white, C—H⋯O by black and π-inter­actions by dark-yellow dashed lines. Distances are given in Å. [Symmetry codes: (i) −*x* + 1, −*y* + 1,-*z*; (viii) *x*, *y* + 1, *z*; (xi) *x* + 1, *y* + 1, *z*.]

In the crystal structure, numerous non-covalant inter­actions are observed. The two nitrate anions are linked *via* O—H⋯O, C—H⋯O and partly *via* N—H⋯O hydrogen bonds with water, phenanthroline and *N*,*N*′-(1,4-phenyl­enedicarbon­yl)diglycine mol­ecules (Fig. 1 ▸ and Table 1 ▸). π–π inter­actions of parallel-displaced phenanthroline ligands and between phenanthroline and *N*,*N*′-(1,4-phenyl­enedicarbon­yl)diglycine solvent mol­ecules stack these components along the *c* axis (Fig. 2 ▸). The centroid-to-centroid distance of *Cg*1⋯*Cg*5 is 3.7094 (8) Å and between *Cg*7⋯*Cg*7 is 3.9973 (9) Å (Fig. 3 ▸), where *Cg*1, *Cg*5 and *Cg*7 are the centroids defined by the ring atoms C37–C39/C37′–C39′, N3/C13-C16/C24 and N4/C19-C23, respectively. These distances are in expected ranges (Barceló-Oliver *et al.*, 2010 ▸; Kumar Seth *et al.*, 2010 ▸). In addition, a T-shaped motif between aromatic rings give rise to C—H⋯π inter­actions and leads to an expected distance (Brandl *et al.*, 2001 ▸; Gathergood *et al.*, 2003 ▸; Horiguchi *et al.*, 2007 ▸; Meyer *et al.*, 2003 ▸; Salonen *et al.*, 2011 ▸) between H20(*Cg*7)⋯*Cg*8 of 3.037 (1) Å, where *Cg*8 is the centroid defined by the ring atoms N5/C25–C28/C36. Moreover, a relatively short N—H⋯π distance of 4.08 (6) Å is observed (Fig. 3 ▸) that is comparable to reference values (Steiner & Koellner, 2001 ▸). Besides the previously mentioned forces, lone-pair⋯π and anion⋯π inter­actions (Fig. 4 ▸) contribute to the consolidation of the supra­molecular network. The lone-pair⋯π inter­actions between the O3 atom of the carb­oxy­lic acid function of the *N*,*N*′-(1,4-phenyl­enedicarbon­yl)diglycine solvent and the *Cg*2 centroid of a phenanthroline ligand are associated with a distance of 3.400 (5) Å. Similar distances of 3.461 (5) Å prevail between the O10 atom of a water mol­ecule and the *Cg*3 centroid of a phenanthroline ligand, where *Cg*2 and *Cg*3 are the centroids defined by the ring atoms N1/C1–C4/C12 and C4–C7/C11/C12, respectively. The values are similar to those found in the literature (Egli & Sarkhel, 2007 ▸; Gao *et al.*, 2009 ▸; Jain *et al.*, 2009 ▸; Mooibroek *et al.*, 2008 ▸; Wan *et al.*, 2008 ▸). Finally, the anion⋯π inter­actions of the nitrate (N9/O4-O6) and *Cg*7 of a phenanthroline ligand are reflected by a distance of 3.628 (4) Å that is comparable to previously reported structures (Ballester, 2008 ▸; Gamez *et al.*, 2007 ▸; Schottel *et al.*, 2008 ▸).

## Database survey   

A search in the Cambridge Structural Database (Version 5.35, November 2013 with three updates; Groom & Allen, 2014 ▸) for crystal structures containing the ligand *N*,*N*′-(1,4-phenyl­enedicarbon­yl)diglycine resulted in six metal-organic compounds (Duan *et al.*, 2010 ▸; Kostakis *et al.*, 2005 ▸, 2011 ▸; Zhang *et al.*, 2005 ▸, 2006 ▸). Some of these structures are composed of inter­penetrating networks. Among them is a structure which includes bi­pyridine besides *N*,*N*′-(1,4-phenyl­enedicarbon­yl)diglycine and shows a number of non-classical inter­actions (Pook *et al.*, 2014 ▸).

## Synthesis and crystallization   

The starting material, *N*,*N*′-(1,4-phenyl­enedicarbon­yl)diglycine, was prepared by the method of Cleaver & Pratt (1955 ▸). Cesium carbonate (2 mmol), 1,10-phenanthroline (1 mmol) and 2,2′-(benzene-1,4-dicarboxamido)­diacetatic acid (1 mmol) were dissolved in a 1:1 (*v*/*v*) mixture of water and methanol (50 ml) and refluxed for 30 minutes. The mixture was allowed to cool to room temperature, and a previously prepared aqueous solution of cobalt nitrate (1 mmol) was slowly added under continuous stirring. Deep dark-orange block-shaped crystals of the title compound were obtained by slow evaporation at room temperature.

## Refinement details   

Crystal data, data collection and structure refinement details are summarized in Table 2 ▸. All C-bound H atoms were positioned with idealized geometry and refined with *U*_iso_(H) = 1.2 *U*_eq_(C) and C—H(aromatic) = 0.94 Å and C—H(methyl­ene) = 0.98 Å using a riding model. The water H atoms were located in a different Fourier map and were refined with O—H distances restrained to 0.82–0.87 Å and with *U*_iso_(H) = 1.5*U*_eq_(O).

## Supplementary Material

Crystal structure: contains datablock(s) I, New_Global_Publ_Block. DOI: 10.1107/S2056989015013006/wm5178sup1.cif

Structure factors: contains datablock(s) I. DOI: 10.1107/S2056989015013006/wm5178Isup2.hkl

Supporting information file. DOI: 10.1107/S2056989015013006/wm5178Isup4.cdx

CCDC reference: 1410901

Additional supporting information:  crystallographic information; 3D view; checkCIF report

## Figures and Tables

**Table 1:** Hydrogen-bond geometry (, )

*D*H*A*	*D*H	H*A*	*D* *A*	*D*H*A*
O3H31O10	0.82(1)	1.80(2)	2.598(5)	162(7)
O10H10*A*O9	0.87(2)	2.09(2)	2.954(6)	172(5)
O10H10*A*O7	0.87(2)	2.54(4)	3.190(7)	132(5)
O10H10*B*O2^i^	0.82(1)	2.07(2)	2.878(6)	171(6)
O11H11*A*O8	0.85(8)	2.32(8)	3.098(9)	154(7)
O11H11*A*O7	0.85(8)	2.53(8)	3.313(8)	154(7)
O11H11*B*O6	0.93(8)	2.02(8)	2.917(7)	162(7)
O12H12*A*O11^ii^	0.88(2)	2.18(7)	2.945(10)	146(11)
O12H12*B*O13	0.88(2)	1.93(5)	2.766(8)	156(11)
O13H13*A*O5^iii^	0.89(2)	1.98(5)	2.827(9)	160(12)
O13H13*B*O12^iv^	0.88(2)	2.09(9)	2.843(11)	143(12)
N7H7O4	0.82(2)	2.06(3)	2.861(6)	165(7)
C3H3O1	0.94	2.37	3.111(6)	135
C33H33O9	0.94	2.54	3.314(7)	140
C38H38O4	0.94	2.47	3.386(7)	166
C41H41*B*O6	0.98	2.67	3.409(7)	132

Symmetry codes: (i) 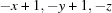; (ii) 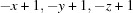; (iii) 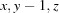; (iv) 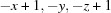.

**Table 2:** Experimental details

Crystal data	
Chemical formula	[Co(C_12_H_8_N_2_)_3_]_2_(NO_3_)_4_C_12_H_12_N_2_O_6_8H_2_O
*M*_r_	1871.48
Crystal system, space group	Triclinic, *P* 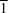
Temperature (K)	223
*a*, *b*, *c* ()	10.6663(18), 14.314(2), 14.573(3)
, , ()	85.403(13), 73.421(14), 82.020(12)
*V* (^3^)	2109.8(6)
*Z*	1
Radiation type	Mo *K*
(mm^1^)	0.49
Crystal size (mm)	0.25 0.23 0.15

Data collection	
Diffractometer	Stoe IPDS 2
Absorption correction	Numerical (*X-AREA*; Stoe, 2008 ▸)
*T*_min_, *T*_max_	0.819, 0.961
No. of measured, independent and observed [*I* > 2(*I*)] reflections	21841, 7956, 4701
*R*_int_	0.138
(sin /)_max_ (^1^)	0.610

Refinement	
*R*[*F* ^2^ > 2(*F* ^2^)], *wR*(*F* ^2^), *S*	0.074, 0.154, 1.05
No. of reflections	7956
No. of parameters	621
No. of restraints	8
H-atom treatment	H atoms treated by a mixture of independent and constrained refinement
_max_, _min_ (e ^3^)	0.43, 0.49

Computer programs: *X-AREA* (Stoe Cie, 2008 ▸), *SHELXS97* and *SHELXL97* (Sheldrick, 2008 ▸), *DIAMOND* (Brandenburg, 2007 ▸), *PLATON* (Spek, 2009 ▸) and *publCIF* (Westrip, 2010 ▸).

## supplementary crystallographic information

### Crystal data

**Table:**

[Co(C_12_H_8_N_2_)_3_]_2_(NO_3_)_4_·C_12_H_12_N_2_O_6_·8H_2_O	*Z* = 1
*M**_r_* = 1871.48	*F*(000) = 968.0
Triclinic, *P*1	*D*_x_ = 1.473 Mg m^−^^3^
Hall symbol: -P 1	Mo *K*α radiation, λ = 0.71073 Å
*a* = 10.6663 (18) Å	Cell parameters from 7956 reflections
*b* = 14.314 (2) Å	θ = 1.0–25.7°
*c* = 14.573 (3) Å	µ = 0.49 mm^−^^1^
α = 85.403 (13)°	*T* = 223 K
β = 73.421 (14)°	Block, orange
γ = 82.020 (12)°	0.25 × 0.23 × 0.15 mm
*V* = 2109.8 (6) Å^3^	

### Data collection

**Table:**

Stoe IPDS 2 diffractometer	7956 independent reflections
Radiation source: fine-focus sealed tube	4701 reflections with *I* > 2σ(*I*)
Graphite monochromator	*R*_int_ = 0.138
ω–scans	θ_max_ = 25.7°, θ_min_ = 2.0°
Absorption correction: numerical (*X-AREA*; Stoe, 2008)	*h* = −13→13
*T*_min_ = 0.819, *T*_max_ = 0.961	*k* = −17→17
21841 measured reflections	*l* = −17→17

### Refinement

**Table:**

Refinement on *F*^2^	Primary atom site location: structure-invariant direct methods
Least-squares matrix: full	Secondary atom site location: difference Fourier map
*R*[*F*^2^ > 2σ(*F*^2^)] = 0.074	Hydrogen site location: inferred from neighbouring sites
*wR*(*F*^2^) = 0.154	H atoms treated by a mixture of independent and constrained refinement
*S* = 1.05	*w* = 1/[σ^2^(*F*_o_^2^) + (0.0452*P*)^2^ + 0.3752*P*] where *P* = (*F*_o_^2^ + 2*F*_c_^2^)/3
7956 reflections	(Δ/σ)_max_ = 0.001
621 parameters	Δρ_max_ = 0.43 e Å^−^^3^
8 restraints	Δρ_min_ = −0.48 e Å^−^^3^

### Special details

**Table:**

Geometry. All e.s.d.'s (except the e.s.d. in the dihedral angle between two l.s. planes) are estimated using the full covariance matrix. The cell e.s.d.'s are taken into account individually in the estimation of e.s.d.'s in distances, angles and torsion angles; correlations between e.s.d.'s in cell parameters are only used when they are defined by crystal symmetry. An approximate (isotropic) treatment of cell e.s.d.'s is used for estimating e.s.d.'s involving l.s. planes.
Refinement. Refinement of *F*^2^ against ALL reflections. The weighted *R*-factor *wR* and goodness of fit *S* are based on *F*^2^, conventional *R*-factors *R* are based on *F*, with *F* set to zero for negative *F*^2^. The threshold expression of *F*^2^ > σ(*F*^2^) is used only for calculating *R*-factors(gt) *etc*. and is not relevant to the choice of reflections for refinement. *R*-factors based on *F*^2^ are statistically about twice as large as those based on *F*, and *R*- factors based on ALL data will be even larger.

### Fractional atomic coordinates and isotropic or equivalent isotropic displacement parameters (Å^2^)

**Table:**

	*x*	*y*	*z*	*U*_iso_*/*U*_eq_	
Co	0.04386 (7)	0.23362 (5)	0.27813 (5)	0.03230 (18)	
O1	−0.0043 (5)	0.7504 (2)	0.0769 (4)	0.0732 (15)	
O2	0.3407 (4)	0.6495 (3)	0.0417 (3)	0.0512 (9)	
O3	0.2889 (4)	0.5625 (2)	0.1806 (3)	0.0471 (9)	
H31	0.342 (5)	0.519 (3)	0.154 (4)	0.071*	
O4	0.3317 (4)	0.9176 (3)	0.1619 (4)	0.0686 (13)	
O5	0.4745 (6)	0.9491 (4)	0.2281 (4)	0.1008 (19)	
O6	0.4044 (5)	0.8125 (4)	0.2524 (4)	0.0841 (15)	
O7	0.5194 (5)	0.5579 (4)	0.2754 (4)	0.0834 (15)	
O8	0.6586 (5)	0.5272 (4)	0.3545 (4)	0.101 (2)	
O9	0.6425 (4)	0.4256 (3)	0.2564 (3)	0.0613 (11)	
O10	0.4710 (4)	0.4210 (3)	0.1299 (3)	0.0468 (9)	
H10A	0.521 (5)	0.428 (4)	0.165 (3)	0.049 (16)*	
H10B	0.528 (5)	0.407 (5)	0.081 (3)	0.07 (2)*	
O11	0.6055 (6)	0.7456 (4)	0.3503 (4)	0.0780 (15)	
H11A	0.604 (7)	0.689 (6)	0.339 (6)	0.09 (3)*	
H11B	0.557 (8)	0.767 (6)	0.307 (6)	0.10 (3)*	
O12	0.5530 (7)	0.1406 (5)	0.4875 (6)	0.114 (2)	
H12A	0.494 (9)	0.151 (9)	0.543 (5)	0.171*	
H12B	0.509 (10)	0.121 (8)	0.451 (7)	0.171*	
O13	0.4193 (7)	0.0330 (5)	0.4067 (6)	0.115 (2)	
H13A	0.453 (11)	0.014 (9)	0.347 (4)	0.172*	
H13B	0.392 (12)	−0.009 (7)	0.454 (6)	0.172*	
N1	0.0324 (4)	0.3664 (3)	0.2003 (3)	0.0368 (10)	
N2	0.2048 (4)	0.2094 (3)	0.1525 (3)	0.0349 (9)	
N3	−0.1049 (4)	0.1827 (3)	0.2298 (3)	0.0387 (10)	
N4	0.0544 (4)	0.0889 (3)	0.3308 (3)	0.0384 (10)	
N5	−0.0900 (4)	0.2861 (3)	0.4089 (3)	0.0354 (9)	
N6	0.1757 (4)	0.2817 (3)	0.3482 (3)	0.0360 (9)	
N7	0.1534 (5)	0.8007 (3)	0.1288 (3)	0.0419 (10)	
H7	0.201 (6)	0.840 (4)	0.130 (5)	0.08 (2)*	
N8	0.6071 (4)	0.5025 (4)	0.2970 (3)	0.0479 (11)	
N9	0.4026 (5)	0.8936 (4)	0.2152 (4)	0.0558 (13)	
C1	−0.0530 (5)	0.4428 (3)	0.2256 (4)	0.0406 (12)	
H1	−0.1207	0.4403	0.2832	0.049*	
C2	−0.0458 (6)	0.5276 (4)	0.1695 (4)	0.0484 (14)	
H2	−0.1077	0.5807	0.1891	0.058*	
C3	0.0531 (6)	0.5313 (4)	0.0858 (4)	0.0485 (14)	
H3	0.0591	0.5875	0.0477	0.058*	
C4	0.1455 (5)	0.4523 (3)	0.0564 (4)	0.0395 (12)	
C5	0.2511 (6)	0.4493 (4)	−0.0297 (4)	0.0515 (15)	
H5	0.2589	0.5029	−0.0717	0.062*	
C6	0.3392 (6)	0.3729 (4)	−0.0528 (4)	0.0527 (15)	
H6	0.4086	0.3742	−0.1094	0.063*	
C7	0.3288 (5)	0.2884 (4)	0.0086 (4)	0.0426 (12)	
C8	0.4200 (5)	0.2073 (4)	−0.0091 (4)	0.0521 (14)	
H8	0.4932	0.2057	−0.0635	0.062*	
C9	0.4024 (6)	0.1303 (4)	0.0529 (4)	0.0542 (15)	
H9	0.4634	0.0754	0.0420	0.065*	
C10	0.2927 (5)	0.1345 (4)	0.1325 (4)	0.0430 (12)	
H10	0.2807	0.0809	0.1743	0.052*	
C11	0.2233 (5)	0.2870 (3)	0.0916 (3)	0.0326 (10)	
C12	0.1301 (5)	0.3703 (3)	0.1167 (3)	0.0342 (11)	
C13	−0.1832 (5)	0.2285 (4)	0.1819 (4)	0.0464 (13)	
H13	−0.1734	0.2921	0.1637	0.056*	
C14	−0.2807 (6)	0.1887 (4)	0.1562 (5)	0.0564 (15)	
H14	−0.3334	0.2244	0.1210	0.068*	
C15	−0.2969 (6)	0.0969 (5)	0.1837 (5)	0.0617 (17)	
H15	−0.3618	0.0684	0.1679	0.074*	
C16	−0.2172 (6)	0.0455 (4)	0.2353 (4)	0.0536 (15)	
C17	−0.2252 (7)	−0.0525 (5)	0.2653 (5)	0.0682 (19)	
H17	−0.2886	−0.0843	0.2514	0.082*	
C18	−0.1424 (7)	−0.0990 (4)	0.3130 (5)	0.0673 (19)	
H18	−0.1496	−0.1629	0.3313	0.081*	
C19	−0.0449 (6)	−0.0555 (4)	0.3367 (4)	0.0500 (14)	
C20	0.0441 (6)	−0.1004 (4)	0.3847 (4)	0.0574 (15)	
H20	0.0426	−0.1648	0.4028	0.069*	
C21	0.1334 (6)	−0.0522 (4)	0.4060 (4)	0.0538 (15)	
H21	0.1931	−0.0827	0.4386	0.065*	
C22	0.1340 (5)	0.0449 (4)	0.3776 (4)	0.0483 (13)	
H22	0.1940	0.0788	0.3932	0.058*	
C23	−0.0350 (5)	0.0412 (3)	0.3087 (4)	0.0413 (12)	
C24	−0.1207 (5)	0.0913 (3)	0.2575 (4)	0.0403 (12)	
C25	−0.2189 (5)	0.2880 (4)	0.4380 (4)	0.0496 (14)	
H25	−0.2604	0.2593	0.4007	0.060*	
C26	−0.2979 (6)	0.3309 (4)	0.5225 (4)	0.0554 (15)	
H26	−0.3896	0.3299	0.5414	0.066*	
C27	−0.2384 (6)	0.3743 (4)	0.5767 (4)	0.0504 (14)	
H27	−0.2895	0.4050	0.6322	0.060*	
C28	−0.1017 (5)	0.3724 (3)	0.5489 (3)	0.0387 (11)	
C29	−0.0309 (6)	0.4149 (4)	0.6016 (4)	0.0456 (13)	
H29	−0.0781	0.4458	0.6580	0.055*	
C30	0.1008 (6)	0.4115 (4)	0.5725 (4)	0.0466 (13)	
H30	0.1441	0.4385	0.6098	0.056*	
C31	0.1770 (5)	0.3674 (3)	0.4854 (4)	0.0414 (12)	
C32	0.3140 (6)	0.3644 (4)	0.4507 (4)	0.0504 (14)	
H32	0.3614	0.3919	0.4847	0.061*	
C33	0.3780 (5)	0.3213 (4)	0.3674 (4)	0.0522 (14)	
H33	0.4700	0.3191	0.3429	0.063*	
C34	0.3053 (5)	0.2801 (4)	0.3186 (4)	0.0439 (12)	
H34	0.3511	0.2497	0.2616	0.053*	
C35	0.1110 (5)	0.3250 (3)	0.4311 (3)	0.0348 (11)	
C36	−0.0303 (5)	0.3275 (3)	0.4638 (3)	0.0347 (11)	
C37	−0.0689 (6)	0.9267 (4)	−0.0025 (4)	0.0510 (15)	
H37	−0.1166	0.8767	−0.0044	0.061*	
C38	0.1019 (6)	0.9861 (4)	0.0429 (5)	0.0528 (15)	
H38	0.1712	0.9774	0.0719	0.063*	
C39	0.0323 (5)	0.9110 (3)	0.0405 (4)	0.0429 (13)	
C40	0.0587 (6)	0.8137 (3)	0.0836 (4)	0.0450 (13)	
C41	0.1763 (5)	0.7131 (3)	0.1820 (4)	0.0424 (12)	
H41A	0.0929	0.6861	0.2063	0.051*	
H41B	0.2040	0.7272	0.2374	0.051*	
C42	0.2788 (5)	0.6397 (3)	0.1250 (4)	0.0371 (11)	

### Atomic displacement parameters (Å^2^)

**Table:**

	*U*^11^	*U*^22^	*U*^33^	*U*^12^	*U*^13^	*U*^23^
Co	0.0358 (4)	0.0300 (3)	0.0299 (3)	−0.0081 (3)	−0.0054 (3)	−0.0008 (3)
O1	0.101 (4)	0.026 (2)	0.121 (4)	−0.015 (2)	−0.076 (3)	0.010 (2)
O2	0.061 (3)	0.050 (2)	0.038 (2)	−0.0048 (18)	−0.0070 (19)	−0.0001 (17)
O3	0.058 (2)	0.033 (2)	0.049 (2)	0.0051 (17)	−0.0179 (19)	0.0010 (17)
O4	0.074 (3)	0.043 (2)	0.105 (4)	−0.004 (2)	−0.053 (3)	−0.002 (2)
O5	0.107 (4)	0.111 (4)	0.114 (5)	−0.058 (4)	−0.057 (4)	0.000 (4)
O6	0.098 (4)	0.073 (3)	0.084 (4)	−0.015 (3)	−0.037 (3)	0.028 (3)
O7	0.074 (3)	0.102 (4)	0.063 (3)	0.021 (3)	−0.017 (3)	−0.002 (3)
O8	0.078 (3)	0.143 (5)	0.100 (4)	0.009 (3)	−0.047 (3)	−0.069 (4)
O9	0.057 (3)	0.056 (3)	0.068 (3)	−0.011 (2)	−0.006 (2)	−0.017 (2)
O10	0.048 (2)	0.050 (2)	0.042 (2)	−0.0005 (18)	−0.012 (2)	−0.0065 (18)
O11	0.089 (4)	0.070 (3)	0.086 (4)	−0.027 (3)	−0.036 (3)	0.006 (3)
O12	0.122 (6)	0.113 (5)	0.123 (6)	−0.050 (4)	−0.040 (4)	−0.019 (4)
O13	0.109 (5)	0.108 (5)	0.149 (7)	0.004 (4)	−0.068 (5)	−0.040 (5)
N1	0.045 (2)	0.029 (2)	0.039 (2)	−0.0075 (18)	−0.013 (2)	−0.0074 (18)
N2	0.041 (2)	0.029 (2)	0.034 (2)	−0.0050 (18)	−0.0084 (18)	−0.0033 (17)
N3	0.042 (2)	0.034 (2)	0.041 (2)	−0.0054 (19)	−0.013 (2)	−0.0021 (19)
N4	0.041 (2)	0.036 (2)	0.038 (2)	−0.0077 (19)	−0.0090 (19)	−0.0002 (18)
N5	0.033 (2)	0.033 (2)	0.039 (2)	−0.0078 (17)	−0.0062 (18)	−0.0014 (17)
N6	0.039 (2)	0.034 (2)	0.033 (2)	−0.0110 (18)	−0.0045 (18)	0.0001 (17)
N7	0.051 (3)	0.029 (2)	0.051 (3)	−0.002 (2)	−0.025 (2)	−0.0001 (19)
N8	0.031 (2)	0.061 (3)	0.049 (3)	−0.011 (2)	−0.004 (2)	−0.006 (2)
N9	0.049 (3)	0.061 (3)	0.058 (3)	−0.006 (2)	−0.014 (3)	−0.011 (3)
C1	0.045 (3)	0.031 (3)	0.047 (3)	−0.001 (2)	−0.015 (2)	−0.007 (2)
C2	0.058 (4)	0.027 (3)	0.069 (4)	0.001 (2)	−0.033 (3)	−0.009 (3)
C3	0.064 (4)	0.035 (3)	0.060 (4)	−0.015 (3)	−0.037 (3)	0.008 (3)
C4	0.054 (3)	0.031 (3)	0.042 (3)	−0.017 (2)	−0.026 (3)	0.012 (2)
C5	0.060 (4)	0.055 (4)	0.047 (3)	−0.028 (3)	−0.024 (3)	0.018 (3)
C6	0.059 (4)	0.065 (4)	0.036 (3)	−0.026 (3)	−0.011 (3)	0.012 (3)
C7	0.043 (3)	0.049 (3)	0.036 (3)	−0.016 (2)	−0.005 (2)	−0.009 (2)
C8	0.045 (3)	0.058 (4)	0.048 (3)	−0.011 (3)	0.004 (3)	−0.020 (3)
C9	0.048 (3)	0.046 (3)	0.059 (4)	0.005 (3)	0.001 (3)	−0.024 (3)
C10	0.044 (3)	0.035 (3)	0.044 (3)	−0.005 (2)	−0.001 (2)	−0.007 (2)
C11	0.036 (3)	0.036 (3)	0.027 (2)	−0.006 (2)	−0.009 (2)	−0.0019 (19)
C12	0.037 (3)	0.034 (3)	0.037 (3)	−0.009 (2)	−0.018 (2)	−0.001 (2)
C13	0.054 (3)	0.040 (3)	0.052 (3)	−0.007 (2)	−0.025 (3)	−0.004 (2)
C14	0.053 (3)	0.058 (4)	0.066 (4)	−0.010 (3)	−0.029 (3)	−0.002 (3)
C15	0.053 (4)	0.067 (4)	0.079 (5)	−0.022 (3)	−0.033 (3)	−0.005 (3)
C16	0.063 (4)	0.046 (3)	0.061 (4)	−0.023 (3)	−0.023 (3)	−0.002 (3)
C17	0.069 (4)	0.055 (4)	0.093 (5)	−0.030 (3)	−0.030 (4)	−0.001 (4)
C18	0.084 (5)	0.040 (3)	0.079 (5)	−0.031 (3)	−0.017 (4)	0.004 (3)
C19	0.057 (3)	0.036 (3)	0.053 (3)	−0.007 (3)	−0.009 (3)	0.001 (3)
C20	0.075 (4)	0.035 (3)	0.052 (4)	−0.007 (3)	−0.005 (3)	0.011 (3)
C21	0.060 (4)	0.045 (3)	0.047 (3)	0.003 (3)	−0.007 (3)	0.007 (3)
C22	0.047 (3)	0.051 (3)	0.045 (3)	−0.003 (3)	−0.014 (3)	0.007 (3)
C23	0.047 (3)	0.027 (3)	0.046 (3)	−0.012 (2)	−0.005 (2)	0.003 (2)
C24	0.046 (3)	0.034 (3)	0.042 (3)	−0.011 (2)	−0.010 (2)	−0.002 (2)
C25	0.046 (3)	0.053 (3)	0.053 (3)	−0.014 (3)	−0.013 (3)	−0.006 (3)
C26	0.038 (3)	0.065 (4)	0.053 (4)	−0.008 (3)	0.005 (3)	−0.004 (3)
C27	0.060 (4)	0.049 (3)	0.036 (3)	−0.004 (3)	−0.003 (3)	−0.004 (2)
C28	0.044 (3)	0.037 (3)	0.029 (3)	−0.004 (2)	0.000 (2)	0.002 (2)
C29	0.070 (4)	0.038 (3)	0.028 (3)	−0.003 (3)	−0.014 (3)	−0.005 (2)
C30	0.057 (4)	0.046 (3)	0.038 (3)	−0.004 (3)	−0.016 (3)	−0.005 (2)
C31	0.054 (3)	0.039 (3)	0.037 (3)	−0.013 (2)	−0.020 (3)	0.004 (2)
C32	0.054 (3)	0.054 (3)	0.050 (3)	−0.015 (3)	−0.022 (3)	0.003 (3)
C33	0.038 (3)	0.071 (4)	0.050 (4)	−0.016 (3)	−0.011 (3)	−0.002 (3)
C34	0.041 (3)	0.059 (3)	0.032 (3)	−0.010 (2)	−0.007 (2)	−0.002 (2)
C35	0.046 (3)	0.029 (2)	0.031 (3)	−0.008 (2)	−0.013 (2)	0.0037 (19)
C36	0.046 (3)	0.027 (2)	0.032 (3)	−0.006 (2)	−0.012 (2)	0.0026 (19)
C37	0.068 (4)	0.028 (3)	0.069 (4)	−0.009 (3)	−0.037 (3)	−0.002 (3)
C38	0.070 (4)	0.029 (3)	0.076 (4)	−0.002 (3)	−0.049 (3)	0.001 (3)
C39	0.060 (3)	0.027 (3)	0.049 (3)	−0.002 (2)	−0.029 (3)	−0.001 (2)
C40	0.056 (3)	0.028 (3)	0.056 (3)	−0.004 (2)	−0.025 (3)	0.002 (2)
C41	0.051 (3)	0.037 (3)	0.039 (3)	0.000 (2)	−0.014 (2)	0.001 (2)
C42	0.043 (3)	0.030 (3)	0.044 (3)	−0.003 (2)	−0.022 (2)	−0.003 (2)

### Geometric parameters (Å, º)

**Table:**

Co—N2	2.137 (4)	C8—C9	1.368 (8)
Co—N1	2.141 (4)	C8—H8	0.9400
Co—N3	2.141 (4)	C9—C10	1.391 (8)
Co—N5	2.148 (4)	C9—H9	0.9400
Co—N4	2.150 (4)	C10—H10	0.9400
Co—N6	2.166 (4)	C11—C12	1.442 (7)
O1—C40	1.226 (6)	C13—C14	1.402 (7)
O2—C42	1.212 (6)	C13—H13	0.9400
O3—C42	1.327 (6)	C14—C15	1.362 (8)
O3—H31	0.822 (10)	C14—H14	0.9400
O4—N9	1.231 (6)	C15—C16	1.389 (8)
O5—N9	1.237 (6)	C15—H15	0.9400
O6—N9	1.241 (6)	C16—C24	1.416 (7)
O7—N8	1.235 (6)	C16—C17	1.442 (8)
O8—N8	1.221 (6)	C17—C18	1.349 (9)
O9—N8	1.251 (6)	C17—H17	0.9400
O10—H10A	0.87 (2)	C18—C19	1.416 (8)
O10—H10B	0.816 (10)	C18—H18	0.9400
O11—H11A	0.85 (8)	C19—C20	1.393 (8)
O11—H11B	0.93 (8)	C19—C23	1.421 (7)
O12—H12A	0.88 (2)	C20—C21	1.365 (8)
O12—H12B	0.88 (2)	C20—H20	0.9400
O13—H13A	0.89 (2)	C21—C22	1.419 (8)
O13—H13B	0.88 (2)	C21—H21	0.9400
N1—C1	1.326 (6)	C22—H22	0.9400
N1—C12	1.361 (6)	C23—C24	1.424 (7)
N2—C10	1.314 (6)	C25—C26	1.411 (8)
N2—C11	1.365 (6)	C25—H25	0.9400
N3—C13	1.312 (6)	C26—C27	1.373 (8)
N3—C24	1.358 (6)	C26—H26	0.9400
N4—C22	1.301 (6)	C27—C28	1.395 (7)
N4—C23	1.368 (6)	C27—H27	0.9400
N5—C25	1.315 (6)	C28—C36	1.408 (7)
N5—C36	1.368 (6)	C28—C29	1.437 (7)
N6—C34	1.323 (6)	C29—C30	1.342 (8)
N6—C35	1.358 (6)	C29—H29	0.9400
N7—C40	1.341 (6)	C30—C31	1.438 (7)
N7—C41	1.449 (6)	C30—H30	0.9400
N7—H7	0.82 (2)	C31—C32	1.400 (8)
N8—O8	1.221 (6)	C31—C35	1.416 (6)
N8—O7	1.235 (6)	C32—C33	1.361 (8)
N8—O9	1.251 (6)	C32—H32	0.9400
N9—O4	1.231 (6)	C33—C34	1.402 (7)
N9—O6	1.241 (6)	C33—H33	0.9400
C1—C2	1.406 (7)	C34—H34	0.9400
C1—H1	0.9400	C35—C36	1.440 (7)
C2—C3	1.369 (8)	C37—C39	1.380 (7)
C2—H2	0.9400	C37—C38^i^	1.381 (7)
C3—C4	1.400 (8)	C37—H37	0.9400
C3—H3	0.9400	C38—C37^i^	1.381 (7)
C4—C12	1.408 (6)	C38—C39	1.397 (7)
C4—C5	1.426 (8)	C38—H38	0.9400
C5—C6	1.337 (8)	C39—C40	1.505 (7)
C5—H5	0.9400	C40—O1	1.226 (6)
C6—C7	1.443 (8)	C41—C42	1.513 (7)
C6—H6	0.9400	C41—H41A	0.9800
C7—C8	1.396 (8)	C41—H41B	0.9800
C7—C11	1.398 (7)		
			
N2—Co—N1	78.29 (16)	C4—C12—C11	119.4 (5)
N2—Co—N3	98.40 (15)	N3—C13—C14	124.3 (5)
N1—Co—N3	93.74 (16)	N3—C13—H13	117.8
N2—Co—N5	165.26 (13)	C14—C13—H13	117.8
N1—Co—N5	94.13 (16)	C15—C14—C13	118.2 (5)
N3—Co—N5	94.67 (15)	C15—C14—H14	120.9
N2—Co—N4	94.91 (16)	C13—C14—H14	120.9
N1—Co—N4	168.75 (15)	C14—C15—C16	119.8 (5)
N3—Co—N4	78.27 (15)	C14—C15—H15	120.1
N5—Co—N4	94.40 (16)	C16—C15—H15	120.1
N2—Co—N6	89.53 (15)	C15—C16—C24	118.1 (5)
N1—Co—N6	90.78 (14)	C15—C16—C17	123.6 (5)
N3—Co—N6	171.53 (17)	C24—C16—C17	118.2 (5)
N5—Co—N6	77.84 (15)	C18—C17—C16	120.6 (5)
N4—Co—N6	98.18 (15)	C18—C17—H17	119.7
C42—O3—H31	114 (5)	C16—C17—H17	119.7
H10A—O10—H10B	98 (6)	C17—C18—C19	122.8 (5)
H11A—O11—H11B	91 (7)	C17—C18—H18	118.6
H12A—O12—H12B	105 (10)	C19—C18—H18	118.6
H13A—O13—H13B	119 (10)	C20—C19—C18	125.1 (5)
C1—N1—C12	118.6 (4)	C20—C19—C23	116.8 (5)
C1—N1—Co	128.1 (4)	C18—C19—C23	118.1 (5)
C12—N1—Co	113.2 (3)	C21—C20—C19	121.1 (5)
C10—N2—C11	117.8 (4)	C21—C20—H20	119.5
C10—N2—Co	128.4 (3)	C19—C20—H20	119.5
C11—N2—Co	113.4 (3)	C20—C21—C22	118.4 (5)
C13—N3—C24	117.7 (4)	C20—C21—H21	120.8
C13—N3—Co	129.0 (3)	C22—C21—H21	120.8
C24—N3—Co	113.2 (3)	N4—C22—C21	122.4 (5)
C22—N4—C23	119.7 (4)	N4—C22—H22	118.8
C22—N4—Co	128.0 (4)	C21—C22—H22	118.8
C23—N4—Co	112.3 (3)	N4—C23—C19	121.7 (5)
C25—N5—C36	118.1 (4)	N4—C23—C24	118.3 (4)
C25—N5—Co	128.3 (3)	C19—C23—C24	120.0 (5)
C36—N5—Co	113.4 (3)	N3—C24—C16	121.8 (5)
C34—N6—C35	117.5 (4)	N3—C24—C23	117.8 (4)
C34—N6—Co	129.4 (3)	C16—C24—C23	120.3 (5)
C35—N6—Co	112.9 (3)	N5—C25—C26	123.2 (5)
C40—N7—C41	121.0 (4)	N5—C25—H25	118.4
C40—N7—H7	125 (5)	C26—C25—H25	118.4
C41—N7—H7	114 (5)	C27—C26—C25	118.9 (5)
O8—N8—O7	118.1 (5)	C27—C26—H26	120.6
O8—N8—O7	118.1 (5)	C25—C26—H26	120.6
O8—N8—O7	118.1 (5)	C26—C27—C28	119.6 (5)
O8—N8—O7	118.1 (5)	C26—C27—H27	120.2
O8—N8—O9	123.1 (5)	C28—C27—H27	120.2
O8—N8—O9	123.1 (5)	C27—C28—C36	117.9 (5)
O7—N8—O9	118.7 (5)	C27—C28—C29	123.4 (5)
O7—N8—O9	118.7 (5)	C36—C28—C29	118.7 (5)
O8—N8—O9	123.1 (5)	C30—C29—C28	121.7 (5)
O8—N8—O9	123.1 (5)	C30—C29—H29	119.2
O7—N8—O9	118.7 (5)	C28—C29—H29	119.2
O7—N8—O9	118.7 (5)	C29—C30—C31	121.2 (5)
O4—N9—O5	119.7 (6)	C29—C30—H30	119.4
O4—N9—O5	119.7 (6)	C31—C30—H30	119.4
O4—N9—O6	119.2 (5)	C32—C31—C35	117.8 (5)
O4—N9—O6	119.2 (5)	C32—C31—C30	123.4 (5)
O5—N9—O6	121.0 (6)	C35—C31—C30	118.9 (5)
O4—N9—O6	119.2 (5)	C33—C32—C31	119.3 (5)
O4—N9—O6	119.2 (5)	C33—C32—H32	120.3
O5—N9—O6	121.0 (6)	C31—C32—H32	120.3
N1—C1—C2	122.4 (5)	C32—C33—C34	119.2 (5)
N1—C1—H1	118.8	C32—C33—H33	120.4
C2—C1—H1	118.8	C34—C33—H33	120.4
C3—C2—C1	118.7 (5)	N6—C34—C33	123.6 (5)
C3—C2—H2	120.6	N6—C34—H34	118.2
C1—C2—H2	120.6	C33—C34—H34	118.2
C2—C3—C4	120.8 (5)	N6—C35—C31	122.5 (5)
C2—C3—H3	119.6	N6—C35—C36	118.0 (4)
C4—C3—H3	119.6	C31—C35—C36	119.5 (5)
C3—C4—C12	116.5 (5)	N5—C36—C28	122.4 (4)
C3—C4—C5	124.7 (5)	N5—C36—C35	117.5 (4)
C12—C4—C5	118.7 (5)	C28—C36—C35	120.1 (4)
C6—C5—C4	122.2 (5)	C39—C37—C38^i^	121.7 (5)
C6—C5—H5	118.9	C39—C37—H37	119.1
C4—C5—H5	118.9	C38^i^—C37—H37	119.1
C5—C6—C7	120.6 (5)	C37^i^—C38—C39	119.9 (5)
C5—C6—H6	119.7	C37^i^—C38—H38	120.0
C7—C6—H6	119.7	C39—C38—H38	120.0
C8—C7—C11	117.4 (5)	C37—C39—C38	118.4 (5)
C8—C7—C6	123.7 (5)	C37—C39—C40	117.4 (4)
C11—C7—C6	118.9 (5)	C38—C39—C40	124.2 (4)
C9—C8—C7	119.8 (5)	O1—C40—N7	122.6 (5)
C9—C8—H8	120.1	O1—C40—N7	122.6 (5)
C7—C8—H8	120.1	O1—C40—C39	120.8 (5)
C8—C9—C10	118.8 (5)	O1—C40—C39	120.8 (5)
C8—C9—H9	120.6	N7—C40—C39	116.7 (4)
C10—C9—H9	120.6	N7—C41—C42	114.7 (4)
N2—C10—C9	123.6 (5)	N7—C41—H41A	108.6
N2—C10—H10	118.2	C42—C41—H41A	108.6
C9—C10—H10	118.2	N7—C41—H41B	108.6
N2—C11—C7	122.6 (5)	C42—C41—H41B	108.6
N2—C11—C12	117.3 (4)	H41A—C41—H41B	107.6
C7—C11—C12	120.0 (4)	O2—C42—O3	125.5 (5)
N1—C12—C4	122.9 (5)	O2—C42—C41	125.5 (4)
N1—C12—C11	117.6 (4)	O3—C42—C41	109.0 (4)
			
N2—Co—N1—C1	−179.5 (4)	Co—N1—C12—C11	1.2 (5)
N3—Co—N1—C1	82.7 (4)	C3—C4—C12—N1	−0.8 (6)
N5—Co—N1—C1	−12.3 (4)	C5—C4—C12—N1	180.0 (4)
N4—Co—N1—C1	127.0 (8)	C3—C4—C12—C11	−178.8 (4)
N6—Co—N1—C1	−90.1 (4)	C5—C4—C12—C11	2.0 (6)
N2—Co—N1—C12	−2.3 (3)	N2—C11—C12—N1	1.6 (6)
N3—Co—N1—C12	−100.1 (3)	C7—C11—C12—N1	−177.4 (4)
N5—Co—N1—C12	164.9 (3)	N2—C11—C12—C4	179.7 (4)
N4—Co—N1—C12	−55.8 (10)	C7—C11—C12—C4	0.7 (6)
N6—Co—N1—C12	87.0 (3)	C24—N3—C13—C14	−1.1 (9)
N1—Co—N2—C10	175.7 (4)	Co—N3—C13—C14	−177.9 (5)
N3—Co—N2—C10	−92.2 (4)	N3—C13—C14—C15	1.0 (10)
N5—Co—N2—C10	115.6 (7)	C13—C14—C15—C16	−0.4 (10)
N4—Co—N2—C10	−13.3 (4)	C14—C15—C16—C24	0.0 (10)
N6—Co—N2—C10	84.8 (4)	C14—C15—C16—C17	−178.4 (7)
N1—Co—N2—C11	3.1 (3)	C15—C16—C17—C18	178.2 (7)
N3—Co—N2—C11	95.2 (3)	C24—C16—C17—C18	−0.2 (10)
N5—Co—N2—C11	−57.0 (8)	C16—C17—C18—C19	0.4 (12)
N4—Co—N2—C11	174.1 (3)	C17—C18—C19—C20	−179.1 (7)
N6—Co—N2—C11	−87.8 (3)	C17—C18—C19—C23	0.0 (10)
N2—Co—N3—C13	−87.7 (5)	C18—C19—C20—C21	−178.9 (6)
N1—Co—N3—C13	−9.0 (5)	C23—C19—C20—C21	1.9 (9)
N5—Co—N3—C13	85.5 (5)	C19—C20—C21—C22	−0.2 (9)
N4—Co—N3—C13	179.0 (5)	C23—N4—C22—C21	0.9 (8)
N2—Co—N3—C24	95.3 (4)	Co—N4—C22—C21	−178.0 (4)
N1—Co—N3—C24	174.0 (4)	C20—C21—C22—N4	−1.3 (9)
N5—Co—N3—C24	−91.5 (4)	C22—N4—C23—C19	0.9 (8)
N4—Co—N3—C24	2.0 (4)	Co—N4—C23—C19	−180.0 (4)
N2—Co—N4—C22	80.9 (5)	C22—N4—C23—C24	180.0 (5)
N1—Co—N4—C22	133.1 (8)	Co—N4—C23—C24	−0.9 (6)
N3—Co—N4—C22	178.4 (5)	C20—C19—C23—N4	−2.3 (8)
N5—Co—N4—C22	−87.7 (5)	C18—C19—C23—N4	178.4 (6)
N6—Co—N4—C22	−9.4 (5)	C20—C19—C23—C24	178.7 (5)
N2—Co—N4—C23	−98.2 (4)	C18—C19—C23—C24	−0.6 (8)
N1—Co—N4—C23	−45.9 (10)	C13—N3—C24—C16	0.6 (8)
N3—Co—N4—C23	−0.6 (3)	Co—N3—C24—C16	178.0 (4)
N5—Co—N4—C23	93.3 (4)	C13—N3—C24—C23	179.4 (5)
N6—Co—N4—C23	171.6 (4)	Co—N3—C24—C23	−3.2 (6)
N2—Co—N5—C25	148.5 (6)	C15—C16—C24—N3	−0.1 (9)
N1—Co—N5—C25	90.2 (5)	C17—C16—C24—N3	178.4 (6)
N3—Co—N5—C25	−3.9 (5)	C15—C16—C24—C23	−178.9 (6)
N4—Co—N5—C25	−82.5 (5)	C17—C16—C24—C23	−0.4 (9)
N6—Co—N5—C25	−179.9 (5)	N4—C23—C24—N3	2.9 (7)
N2—Co—N5—C36	−27.1 (8)	C19—C23—C24—N3	−178.1 (5)
N1—Co—N5—C36	−85.4 (3)	N4—C23—C24—C16	−178.3 (5)
N3—Co—N5—C36	−179.5 (3)	C19—C23—C24—C16	0.8 (8)
N4—Co—N5—C36	101.9 (3)	C36—N5—C25—C26	0.3 (8)
N6—Co—N5—C36	4.5 (3)	Co—N5—C25—C26	−175.1 (4)
N2—Co—N6—C34	−7.2 (4)	N5—C25—C26—C27	1.0 (9)
N1—Co—N6—C34	−85.5 (4)	C25—C26—C27—C28	−2.0 (9)
N5—Co—N6—C34	−179.6 (5)	C26—C27—C28—C36	1.6 (8)
N4—Co—N6—C34	87.7 (4)	C26—C27—C28—C29	−179.3 (5)
N2—Co—N6—C35	167.8 (3)	C27—C28—C29—C30	180.0 (5)
N1—Co—N6—C35	89.5 (3)	C36—C28—C29—C30	−0.9 (8)
N5—Co—N6—C35	−4.5 (3)	C28—C29—C30—C31	1.8 (8)
N4—Co—N6—C35	−97.3 (3)	C29—C30—C31—C32	178.2 (5)
O8—O8—N8—O7	0.0 (3)	C29—C30—C31—C35	−1.5 (8)
O8—O8—N8—O7	0.0 (3)	C35—C31—C32—C33	−0.1 (8)
O8—O8—N8—O9	0.0 (5)	C30—C31—C32—C33	−179.8 (5)
O8—O8—N8—O9	0.0 (5)	C31—C32—C33—C34	−0.5 (9)
O7—O7—N8—O8	0.0 (3)	C35—N6—C34—C33	−0.7 (8)
O7—O7—N8—O8	0.0 (3)	Co—N6—C34—C33	174.2 (4)
O7—O7—N8—O9	0.0 (2)	C32—C33—C34—N6	1.0 (9)
O7—O7—N8—O9	0.0 (2)	C34—N6—C35—C31	−0.1 (7)
O9—O9—N8—O8	0.0 (4)	Co—N6—C35—C31	−175.7 (4)
O9—O9—N8—O8	0.0 (4)	C34—N6—C35—C36	179.7 (4)
O9—O9—N8—O7	0.0 (3)	Co—N6—C35—C36	4.0 (5)
O9—O9—N8—O7	0.0 (3)	C32—C31—C35—N6	0.5 (7)
O4—O4—N9—O5	0.0 (4)	C30—C31—C35—N6	−179.8 (5)
O4—O4—N9—O6	0.00 (14)	C32—C31—C35—C36	−179.3 (4)
O4—O4—N9—O6	0.00 (14)	C30—C31—C35—C36	0.4 (7)
O6—O6—N9—O4	0.0 (6)	C25—N5—C36—C28	−0.7 (7)
O6—O6—N9—O4	0.0 (6)	Co—N5—C36—C28	175.3 (4)
O6—O6—N9—O5	0.0 (8)	C25—N5—C36—C35	180.0 (4)
C12—N1—C1—C2	−0.2 (7)	Co—N5—C36—C35	−3.9 (5)
Co—N1—C1—C2	176.9 (3)	C27—C28—C36—N5	−0.3 (7)
N1—C1—C2—C3	−0.1 (7)	C29—C28—C36—N5	−179.4 (4)
C1—C2—C3—C4	0.0 (7)	C27—C28—C36—C35	179.0 (4)
C2—C3—C4—C12	0.4 (7)	C29—C28—C36—C35	−0.1 (7)
C2—C3—C4—C5	179.6 (5)	N6—C35—C36—N5	−0.1 (6)
C3—C4—C5—C6	177.7 (5)	C31—C35—C36—N5	179.7 (4)
C12—C4—C5—C6	−3.2 (7)	N6—C35—C36—C28	−179.4 (4)
C4—C5—C6—C7	1.5 (8)	C31—C35—C36—C28	0.4 (7)
C5—C6—C7—C8	−177.5 (5)	C38^i^—C37—C39—C38	0.2 (11)
C5—C6—C7—C11	1.3 (7)	C38^i^—C37—C39—C40	179.0 (6)
C11—C7—C8—C9	1.2 (7)	C37^i^—C38—C39—C37	−0.2 (10)
C6—C7—C8—C9	180.0 (5)	C37^i^—C38—C39—C40	−178.9 (6)
C7—C8—C9—C10	0.4 (8)	O1—O1—C40—N7	0.0 (3)
C11—N2—C10—C9	−0.2 (7)	O1—O1—C40—C39	0.00 (10)
Co—N2—C10—C9	−172.6 (4)	C41—N7—C40—O1	−7.1 (9)
C8—C9—C10—N2	−0.9 (9)	C41—N7—C40—O1	−7.1 (9)
C10—N2—C11—C7	1.9 (6)	C41—N7—C40—C39	172.9 (5)
Co—N2—C11—C7	175.4 (3)	C37—C39—C40—O1	2.9 (9)
C10—N2—C11—C12	−177.0 (4)	C38—C39—C40—O1	−178.3 (6)
Co—N2—C11—C12	−3.5 (5)	C37—C39—C40—O1	2.9 (9)
C8—C7—C11—N2	−2.4 (7)	C38—C39—C40—O1	−178.3 (6)
C6—C7—C11—N2	178.7 (4)	C37—C39—C40—N7	−177.2 (6)
C8—C7—C11—C12	176.5 (4)	C38—C39—C40—N7	1.6 (9)
C6—C7—C11—C12	−2.4 (7)	C40—N7—C41—C42	92.4 (6)
C1—N1—C12—C4	0.7 (6)	N7—C41—C42—O2	0.5 (7)
Co—N1—C12—C4	−176.8 (3)	N7—C41—C42—O3	−178.7 (4)
C1—N1—C12—C11	178.7 (4)		

Symmetry code: (i) −*x*, −*y*+2, −*z*.

### Hydrogen-bond geometry (Å, º)

**Table:**

*D*—H···*A*	*D*—H	H···*A*	*D*···*A*	*D*—H···*A*
O3—H31···O10	0.82 (1)	1.80 (2)	2.598 (5)	162 (7)
O10—H10*A*···O9	0.87 (2)	2.09 (2)	2.954 (6)	172 (5)
O10—H10*A*···O7	0.87 (2)	2.54 (4)	3.190 (7)	132 (5)
O10—H10*B*···O2^ii^	0.82 (1)	2.07 (2)	2.878 (6)	171 (6)
O11—H11*A*···O8	0.85 (8)	2.32 (8)	3.098 (9)	154 (7)
O11—H11*A*···O7	0.85 (8)	2.53 (8)	3.313 (8)	154 (7)
O11—H11*B*···O6	0.93 (8)	2.02 (8)	2.917 (7)	162 (7)
O12—H12*A*···O11^iii^	0.88 (2)	2.18 (7)	2.945 (10)	146 (11)
O12—H12*B*···O13	0.88 (2)	1.93 (5)	2.766 (8)	156 (11)
O13—H13*A*···O5^iv^	0.89 (2)	1.98 (5)	2.827 (9)	160 (12)
O13—H13*B*···O12^v^	0.88 (2)	2.09 (9)	2.843 (11)	143 (12)
N7—H7···O4	0.82 (2)	2.06 (3)	2.861 (6)	165 (7)
C3—H3···O1	0.94	2.37	3.111 (6)	135
C33—H33···O9	0.94	2.54	3.314 (7)	140
C38—H38···O4	0.94	2.47	3.386 (7)	166
C41—H41*B*···O6	0.98	2.67	3.409 (7)	132

Symmetry codes: (ii) −*x*+1, −*y*+1, −*z*; (iii) −*x*+1, −*y*+1, −*z*+1; (iv) *x*, *y*−1, *z*; (v) −*x*+1, −*y*, −*z*+1.

## References

[bb1] Albrecht, M., Gjikaj, M. & Schmidt, A. (2010). *Tetrahedron*, **66**, 7149–7154.

[bb2] Allendorf, M. D., Bauer, C. A., Bhakta, R. K. & Houk, R. J. T. (2009). *Chem. Soc. Rev.* **38**, 1330–1352. 10.1039/b802352m 19384441

[bb3] Allendorf, M. D., Foster, M. E., Léonard, F., Stavila, V., Feng, P. L., Doty, F. P., Leong, K., Ma, E. Y., Johnston, S. R. & Talin, A. A. (2015). *J. Phys. Chem. Lett.* **6**, 1182–1195. 10.1021/jz5026883 26262970

[bb4] Ballester, P. (2008). In *Anions and π-Aromatic Systems. Do They Interact Attractively?* In *Recognition of Anions*, edited by R. Vilar. Heidelberg, Berlin: Springer.

[bb5] Barceló-Oliver, M., Terrón, A., García-Raso, A., Lah, N. & Turel, I. (2010). *Acta Cryst.* C**66**, o313–o316. 10.1107/S010827011001841X 20522953

[bb6] Brandenburg, K. (2007). *DIAMOND*. Crystal Impact GbR, Bonn, Germany.

[bb7] Brandl, M., Weiss, M. S., Jabs, A., Sühnel, J. & Hilgenfeld, R. (2001). *J. Mol. Biol.* **307**, 357–377. 10.1006/jmbi.2000.4473 11243825

[bb8] Cleaver, C. S. & Pratt, B. C. (1955). *J. Am. Chem. Soc.* **77**, 1544–1546.

[bb9] Cook, T. R., Zheng, Y.-R. & Stang, P. J. (2013). *Chem. Rev.* **113**, 734–777. 10.1021/cr3002824 PMC3764682 23121121

[bb10] Doty, F. P., Bauer, C. A., Skulan, A. J., Grant, P. G. & Allendorf, M. D. (2009). *Adv. Mater.* **21**, 95–101.

[bb11] Duan, J., Zheng, B., Bai, J., Zhang, Q. & Zuo, C. (2010). *Inorg. Chim. Acta*, **363**, 3172–3177.

[bb12] Egli, M. & Sarkhel, S. (2007). *Acc. Chem. Res.* **40**, 197–205. 10.1021/ar068174u 17370991

[bb13] Gamez, P., Mooibroek, T. J., Teat, S. J. & Reedijk, J. (2007). *Acc. Chem. Res.* **40**, 435–444. 10.1021/ar7000099 17439191

[bb14] Gao, X.-L., Lu, L.-P. & Zhu, M.-L. (2009). *Acta Cryst.* C**65**, o123–o127. 10.1107/S0108270109005836 19346605

[bb15] Gathergood, N., Scammells, P. J. & Fallon, G. D. (2003). *Acta Cryst.* C**59**, o485–o487. 10.1107/s0108270103015622 12944652

[bb16] Geraghty, M., McCann, M., Devereux, M. & McKee, V. (1999). *Inorg. Chim. Acta*, **293**, 160–166.

[bb17] Groom, C. R. & Allen, F. H. (2014). *Angew. Chem. Int. Ed.* **53**, 662–671. 10.1002/anie.201306438 24382699

[bb18] Horiguchi, M., Okuhara, S., Shimano, E., Fujimoto, D., Takahashi, H., Tsue, H. & Tamura, R. (2007). *Cryst. Growth Des.* **7**, 1643–1652.

[bb19] Jain, A., Ramanathan, V. & Sankararamakrishnan, R. (2009). *Protein Sci.* **18**, 595–605. 10.1002/pro.67 PMC2760365 19241386

[bb20] Janiak, C. (2000). *J. Chem. Soc. Dalton Trans.* pp. 3885–3896.

[bb21] Kitagawa, S., Kitaura, R. & Noro, S. (2004). *Angew. Chem.* **116**, 2388–2430. 10.1002/anie.200300610 15114565

[bb22] Kostakis, G. E., Casella, L., Boudalis, A. K., Monzani, E. & Plakatouras, J. C. (2011). *New J. Chem.* **35**, 1060–1071.

[bb23] Kostakis, G. E., Casella, L., Hadjiliadis, N., Monzani, E., Kourkoumelis, N. & Plakatouras, J. C. (2005). *Chem. Commun.*, pp. 3859–3861. 10.1039/b502788h 16041441

[bb24] Kumar Seth, S., Dey, B., Kar, T. & Mukhopadhyay, S. (2010). *J. Mol. Struct.* **973**, 81–88.

[bb25] Leong, W. L. & Vittal, J. J. (2011). *Chem. Rev.* **111**, 688–764. 10.1021/cr100160e 20804195

[bb26] Li, L.-M., Li, Y.-F., Liu, L. & Zhang, Z.-H. (2011). *Acta Cryst.* E**67**, m973.

[bb27] Meyer, E. A., Castellano, R. K. & Diederich, F. (2003). *Angew. Chem. Int. Ed.* **42**, 1210–1250. 10.1002/anie.200390319 12645054

[bb28] Mooibroek, T. J., Gamez, P. & Reedijk, J. (2008). *CrystEngComm*, **10**, 1501–1515.

[bb29] Perry IV, J. J., Feng, P. L., Meek, S. T., Leong, K., Doty, F. P. & Allendorf, M. D. (2012). *J. Mater. Chem.* **22**, 10235–10248.

[bb30] Pook, N.-P., Gjikaj, M. & Adam, A. (2013). *Acta Cryst.* E**69**, o1731. 10.1107/S1600536813029632 PMC4004431 24860287

[bb31] Pook, N.-P., Gjikaj, M. & Adam, A. (2014). *Acta Cryst.* E**70**, m160–m161. 10.1107/S160053681400631X PMC4011312 24860299

[bb32] Salonen, L. M., Ellermann, M. & Diederich, F. (2011). *Angew. Chem. Int. Ed.* **50**, 4808–4842. 10.1002/anie.201007560 21538733

[bb33] Schneider, H.-J. (2009). *Angew. Chem. Int. Ed.* **48**, 3924–3977.

[bb34] Schottel, B. L., Chifotides, H. T. & Dunbar, K. R. (2008). *Chem. Soc. Rev.* **37**, 68–83. 10.1039/b614208g 18197334

[bb35] Sheldrick, G. M. (2008). *Acta Cryst.* A**64**, 112–122. 10.1107/S0108767307043930 18156677

[bb36] Spek, A. L. (2009). *Acta Cryst.* D**65**, 148–155. 10.1107/S090744490804362X PMC2631630 19171970

[bb37] Steiner, T. & Koellner, G. (2001). *J. Mol. Biol.* **305**, 535–557. 10.1006/jmbi.2000.4301 11152611

[bb38] Stoe & Cie (2008). *X-AREA.* Stoe & Cie, Darmstadt, Germany.

[bb39] Wan, C.-Q., Chen, X.-D. & Mak, T. C. W. (2008). *CrystEngComm*, **10**, 475–478.

[bb40] Westrip, S. P. (2010). *J. Appl. Cryst.* **43**, 920–925.

[bb41] Yamada, T., Otsubo, K., Makiura, R. & Kitagawa, H. (2013). *Chem. Soc. Rev.* **42**, 6655–6669. 10.1039/c3cs60028a 23817780

[bb42] Zhang, H.-T., Li, Y.-Z., Wang, T.-W., Nfor, E. N., Wang, H.-Q. & You, X.-Z. (2006). *Eur. J. Inorg. Chem.* pp. 3532–3536.

[bb43] Zhang, H.-T. & You, X.-Z. (2005). *Acta Cryst.* E**61**, m1163–m1165.

